# Seventeen-year evaluation of breast cancer screening: the DOM project, The Netherlands. Diagnostisch Onderzoek (investigation) Mammacarcinoom.

**DOI:** 10.1038/bjc.1998.609

**Published:** 1998-10

**Authors:** G. A. Miltenburg, P. H. Peeters, J. Fracheboud, H. J. Collette

**Affiliations:** Julius Centre for Patient Oriented Research, Medical School, Utrecht University, The Netherlands.

## Abstract

The DOM project is a non-randomized population-based breast cancer screening programme in Utrecht which started in 1974-75. The 17-year effect has been evaluated by a case-control study of breast cancer deaths during the period 1975-92 in women living in the city of Utrecht, born between 1911 and 1925, whose breast cancers were diagnosed after the initiation of the DOM project. Controls (three for each case) were defined as women having the same year of birth as the case, living in the city of Utrecht at the time the case died, and having had the opportunity of screening in the DOM project. Screening in the period 1975-92 indicated a breast cancer mortality reduction of 46% (odds ratio of 0.54, 95% confidence interval 0.37-0.79). The strongest protective effect was found at a screening interval of 2 years or less (mortality reduction of 62%, odds ratio of 0.38), and for the highest number of screens (mortality reduction of 68%, odds ratio of 0.32 for more than four screens). Exclusion of breast cancer deaths that occurred within 1 year of diagnosis, to allow for 'lead-time' bias, gave an odds ratio of 0.61. Early diagnosis of breast cancer by screening reduces breast cancer mortality in the long term. Bias due to the study design may slightly overestimate the protective effect. A screening programme with a 2-yearly, or smaller, interval between successive screens will improve the protection of screening.


British Journal of Cancer (1998) 78(7). 962–965
© 1998 Cancer Research Campaign

### Seventeen-year evaluation of breast cancer screening: the DOM project, The Netherlands

**GAJ Miltenburg, PHM Peeters, J Fracheboud and HJA Collette**

Julius Centre for Patient Oriented Research. Medical School. Utrecht University. The Netherlands

**Summary** The DOM project is a non-randomized population-based breast cancer screening programme in Utrecht which started in 1974–75. The 17-year effect has been evaluated by a case–control study of breast cancer deaths during the period 1975–92 in women living in the city of Utrecht, born between 1911 and 1925, whose breast cancers were diagnosed after the initiation of the DOM project. Controls (three for each case) were defined as women having the same year of birth as the case, living in the city of Utrecht at the time the case died, and having had the opportunity of screening in the DOM project. Screening in the period 1975–92 indicated a breast cancer mortality reduction of 46% (odds ratio of 0.54, 95% confidence interval 0.37–0.79). The strongest protective effect was found at a screening interval of 2 years or less (mortality reduction of 62%, odds ratio of 0.38), and for the highest number of screens (mortality reduction of 68%, odds ratio of 0.32 for more than four screens). Exclusion of breast cancer deaths that occurred within 1 year of diagnosis, to allow for 'lead-time' bias, gave an odds ratio of 0.61. Early diagnosis of breast cancer by screening reduces breast cancer mortality in the long term. Bias due to the study design may slightly overestimate the protective effect. A screening programme with a 2-yearly, or smaller, interval between successive screens will improve the protection of screening.

**Keywords:** breast cancer: mammographical screening: long-term evaluation: case–control study

The purpose of the present paper is to evaluate long-term benefits of the breast cancer screening in the DOM project in The Netherlands, by means of a nested case–control study. Evaluation was made of two particular forms of bias to which attention has recently been drawn (Hosek et al. 1996: Weiss and Lazovich. 1996).

### SUBJECTS AND METHODS

The DOM [Diagnostisch Onderzoek (investigation) Mammacarcinoom] project started in December 1974 in the city of Utrecht. Initially, the screening was limited to women aged 50–64 at intake (birth cohort 1911–25), and they were screened by mammography. Of 20 555 eligible women, 14 697 (72%) attended for screening. The intervals between successive screening examinations (screens) were of different length, namely 1, 1½, 2 and 4 years. At the first examination, both mediolateral and craniocaudal projections were obtained: in subsequent examinations, mammography was restricted to the mediolateral projection. A woman who did not participate in the first screening was not invited for the second screening and so on.

Five examinations had been completed by 1984. A breast cancer registry was set up and cooperation with general practitioners, local authorities and the Central Bureau for Statistics (CBS) ensured the follow-up of the invited women. More detailed information about the screening design has been described previously (Collette et al. 1984. 1992: de Waard et al. 1984). From 1985 onwards, the DOM project was gradually integrated in the nationwide breast cancer screening programme in The Netherlands. This programme invites women aged 50–69, at 2-year intervals and independently of preceding participation, to be screened.

In the present study, cases were defined as breast cancer deaths in the period 1975–92 in women living in the city of Utrecht who were born between 1911 and 1925 and whose breast cancers were diagnosed between 1975 and 1992. Information about tumour size, axillary status and mode of detection of the breast cancers at diagnosis were extracted from the DOM project breast cancer registry. Causes of death were provided by general practitioners or hospitals and checked against the breast cancer registry. Controls were defined as women living in the city of Utrecht at the time the case died, and having the same year of birth. Age matching was done because response rate to the screening and breast cancer mortality are age dependent. For each case, three controls were selected at random from the screening invitation file, i.e. from among all women in the 1911–25 cohort who were resident in Utrecht in 1974.

For all cases and controls, the screening history was taken for the time up to and including the date of diagnosis of the case. To evaluate the bias of 'misclassification of exposure' (Hosek et al. 1996) due to inclusion of the diagnostic screening of screen-detected cases that artificially restricts the chances of controls having undergone screening, the analysis was also done excluding these screens from the screening history. A second form of bias, 'lead-time' bias (Weiss and Lazovich. 1996), was evaluated by excluding breast cancer deaths with a short follow-up period after diagnosis (i.e. deaths of patients who were less likely to have been screened), because their inclusion would give the impression of a disproportionately large number of deaths from breast cancer in unscreened women. Maximum likelihood estimation of the odds ratio (OR) associated with breast cancer screening was obtained

*Received 29 September 1997*
*Revised 31 December 1997*
*Accepted 25 March 1998*

*Correspondence to:* PHM Peeters. Julius Centre for Patient Oriented Research. Medical School. Utrecht University. PO Box 85500. 3508 GA Utrecht. The Netherlands

**Table 1** Number of matched case–control pairs, odds ratios and 95% confidence intervals by period of death from breast cancer (exposure defined as at least one screening examination before or at diagnosis of the case)

| Period of death | Case–control pairs | Odds ratio (95% confidence interval) | Years of follow-up | Reference |
|---|---|---|---|---|
| 1975–81 | 46 | 0.30 (0.13–0.70) | 6 | Collette et al. (1984) |
| 1975–83 | 59 | 0.35 (0.17–0.71) | 8 | Waard et al (1986) |
| 1975–87 | 116 | 0.52 (0.32–0.83) | 12 | Collette et al (1992) |
| 1975–92 | 177 | 0.54 (0.37–0.79) | 17 | Current study |

**Table 2** Number of cases and controls, odds ratios, 95% confidence intervals, and percentage of cases and controls screened before or at diagnosis of the case by 5-year birth cohorts (exposure defined as at least one screening examination before or at diagnosis of the case)

| Birth cohort | Cases (% screened) | Controls (% screened) | Odds ratio (95% confidence interval) |
|---|---|---|---|
| 1911–15 | 63 (43) | 189 (60) | 0.40 (0.21–0.75) |
| 1916–20 | 60 (52) | 180 (66) | 0.57 (0.31–1.04) |
| 1921–25 | 54 (59) | 162 (65) | 0.71 (0.34–1.48) |
| 1911-25 (total) | 177 (51) | 531 (64) | 0.54 (0.37–0.79) |

**Table 3** Number of cases and controls, odds ratios and 95% confidence intervals, by interval between the last screen and diagnosis of the case; by number of screens; and by level of participation

| | Cases | Controls | Odds ratio (95% confidence interval) |
|---|---|---|---|
| Interval between last screen and diagnosis of the case | | | |
| No screens | 87 | 193 | 1.00 |
| ≤ 1 year | 29 | 147 | 0.38 (0.22–0.63) |
| 1–2 years | 12 | 63 | 0.38 (0.18–0.77) |
| 2–3 years | 8 | 24 | 0.69 (0.28–1.68) |
| 3–4 years | 8 | 22 | 0.85 (0.32–2.22) |
| > 4 years | 33 | 82 | 0.91 (0.53–1.57) |
| Number of screens before or at diagnosis of the case | | | |
| 0 | 87 | 193 | 1.00 |
| 1 | 27 | 85 | 0.67 (0.40–1.13) |
| 2–4 | 49 | 177 | 0.55 (0.35–0.85) |
| 5–8 | 14 | 76 | 0.32 (0.16–0.64) |
| Participation in all offered screens[a] before or at diagnosis of the case | | | |
| No screens | 87 | 193 | 1.00 |
| Non-compliance[b] | 47 | 129 | 0.73 (0.46–1.16) |
| Full compliance[c] | 43 | 209 | 0.42 (0.27–0.65) |

[a]The number of offered screens before or at diagnosis of the case depends on the date of diagnosis. [b]Women who were screened at least once but who did not take up all screens offered before or at diagnosis of the case. [c]Women who took all offered screens before or at diagnosis of the case.

by means of a conditional logistic regression analysis for matched sets (Breslow and Day, 1980). This measure of effect can be considered as an estimate of the relative risk. The analyses were performed with the statistical package EGRET (Egret, 1990).

### RESULTS

Between 1975 and 1992, a total of 846 breast cancer cases were diagnosed in Utrecht, and for 177 of them it had been the cause of death. Of these 177 breast cancer cases, 13% ($n = 23$) were detected by screening, 12% ($n = 21$) were detected in the interval between two successive screens and 75% ($n = 133$) were not screen detected (62 were among never-attenders). All 177 tumours of the cases who had died were invasive at diagnosis. Most tumours were ≤ 20 mm: 61%, 62% and 36% respectively in the three detection groups.

Of the deceased breast cancer cases, 51% had at least one screening examination, compared with 64% of the control women [OR 0.54, 95% confidence interval 0.37–0.79 (Tables 1 and 2)]. This indicates a 46% reduction in breast cancer mortality reduction among participants of the screening project. ORs from previous analyses of the DOM project (Collette et al, 1984, 1992; de Waard et al, 1986) are also presented in Table 1.

The results of screening on breast cancer mortality in the period 1975–92 stratified by birth cohort are presented in Table 2. The strongest protective effect was found in the eldest birth cohort (1911–15), and this decreased in the two younger birth cohorts. However, the confidence intervals of the ORs are broad and these differences are not statistically significant.

In Table 3, ORs are shown for different intervals between the last screening examination and diagnosis of the case, the number of screens, and participation in all offered screens before or at diagnosis of the case. Women who never participated (87 cases and 193 controls) represented the reference group. The strongest protective effect was found for the interval of 2 years or less between the last screen and diagnosis of the case (OR 0.38, 95% confidence interval 0.18–0.77). The protection of screening decreased with increasing interval periods since the last screen. With regard to the number of screens, the ORs decreased with increasing number of screens before diagnosis of the case. The OR for women who participated in all offered screens was lower than the OR for women without full compliance. In the present case–control study, all screening visits up to, and including, the diagnosis were counted as a positive visit. For the screen-detected cases ($n = 23$), the screening examination, from which the diagnosis was made, was included as part of the screening history. To evaluate the possible bias due to including this examination, an analysis of the same data (177 matched case–control pairs) was performed, excluding the diagnostic screening examination. This gave an OR of 0.38 (95% confidence interval 0.26–0.56), suggesting a higher protective effect of screening.

To evaluate 'lead-time-bias', Table 4 shows the influence of excluding cases with successively longer follow-up times between diagnosis and dying. If this period is less than 1 year, the effect of screening seems to be overestimated.

### DISCUSSION

This case–control approach indicates a 46% reduction in breast cancer mortality after 17 years of follow-up for participants of the screening programme. The strongest protective effect of screening is found in the oldest birth cohort, women of 60–64 years at the start of the project. A higher level of protection in older women has been described earlier in the DOM project (Collette et al, 1984, 1985, 1992) and in other studies (Tabàr et al, 1995). Cancers detected at screening would be expected to be of lower malignant

Table 4 Number of matched case–control pairs, odds ratios and 95% confidence intervals for different lengths of time between diagnosis and death of the case (exposure defined as at least one screening examination before or at diagnosis of the case)

| Minimal period in years between diagnosis and death of the case | Case–control pairs | Odds ratio (95% confidence interval) |
|---|---|---|
| 0 (all cases) | 177 | 0.54 (0.37–0.79) |
| 1 | 151 | 0.61 (0.40–0.92) |
| 2 | 126 | 0.64 (0.41–0.99) |
| 3 | 100 | 0.62 (0.37–1.01) |
| 4 | 79 | 0.56 (0.32–0.97) |
| 5 | 58 | 0.61 (0.32–1.16) |

potential than cancers that were not screen detected. In the present study, 61% of all screen-detected breast cancers were small (≤ 20 mm), a percentage that is in reasonable accord with results of the Dutch national screening programme and the Finnish study (Hakama et al, 1995; Koning et al, 1995*a*). Because the present study concerns breast cancer deaths, it is not surprising that a high percentage (74%) of the screen-detected tumours were axillary positive at diagnosis. For both cases and controls, participation in the DOM project was low. However, some controls, of course, responded to the screening after the pseudo-diagnosis; resulting in higher true attendance rates of 68%, 74% and 78%, respectively, for the three 5-year birth cohorts show in Table 2. Selection bias due to a 'healthy screenee effect' cannot be excluded in this case–control study, because both the number of screenings before diagnosis of the case and compliance show protective effect (Table 3). Two other forms of bias in a case–control design may also be relevant. 'Misclassification of exposure' bias due to including the screening examination, from which the diagnosis was made, in the screening history of the screen-detected cases (Hosek et al, 1996) appears to be present in the current study. Its effect is reflected in an OR of 0.54 (with inclusion of the diagnostic screening; 95% confidence interval 0.37–0.79) and 0.38 (without screening; 95% confidence interval 0.26–0.56). The unbiased OR may be expected to lie between the two estimates, because systematic exclusion of the screening examination can cause bias in the opposite direction to that of its inclusion (Hosek et al, 1996). The other form of bias, 'lead-time bias', seems also to be present in this study. Too short a follow-up time of incident cases leads to an artificially large number of deaths from breast cancer in unscreened cases (in which lead time is absent), resulting in an overestimation of the protective effect (Weiss and Lazovich, 1996). The present study indicates a reduction of breast cancer mortality of 46% because of screening. Exclusion of cases with a follow-up time of less than 1 year reduces this figure to 39%, which is in reasonable agreement with new results from the Swedish trials: a 34% mortality reduction for women aged 50–74 years (Tabàr et al, 1995) and an expected reduction of between 24% and 32% for women aged 50–69 years at trial entry (Koning et al, 1995*b*).

The estimates of the protective effect of the screening on breast cancer mortality in the long term have decreased from a 70% reduction after 6 years' follow-up, to a 48% reduction after 12 years' follow-up, and finally to a 46% reduction (95% confidence interval 21–63) after 17 years follow-up (Table 1). This decrease may be due to the following: first, the screening programme could have had a positive influence on the whole population, including women who were never screened, by promoting awareness of breast cancer and so increasing self-examination and readiness to seek early medical help. Another explanation for the observed decrease in the screening effect could be an improved therapy or a change in aggressiveness of the disease in time. A slight improvement of the prognosis of breast cancer during 1970–74 to 1980–84 in The Netherlands has been reported (Nab, 1995). Furthermore, 'lead-time' bias could have had a larger influence in the shorter follow-up periods of the previously published results of the DOM project, leading to greater overestimation of the protective effect in the earlier periods. Finally, it might be that in the short term the screening partly postpones breast cancer death and partly prevents it (Collette et al, 1984, 1985). However, after 12 and 17 years of follow-up, the protective effect has stabilized, suggesting a real reduction of breast cancer mortality in the long term.

At the moment, the nationwide breast cancer screening programme in The Netherlands invites women at 2-year intervals (Koning et al, 1995*a*), whereas in the United Kingdom women are invited every 3 years (Patnick et al, 1995; Asbury et al, 1996). On the basis of the results of the present study (Table 3), a 2-yearly screening programme seems preferable to a 3-yearly programme. However, these results should be interpreted with caution because of the small number of cases and controls with an interval of 2–3 years between last screen and diagnosis of the case.

In the present study, protective effects of screening were found in shorter screening intervals, after more screens, in older women, and in women who are willing to participate. It was not possible to evaluate a possible interaction between these factors, because of small numbers and their inter-correlation. It is not likely that the protective effect of screening in the oldest 5-year birth cohort is fully attributable to attendance at more screening examinations, because this birth cohort saw the lowest percentage of cases and controls who were screened two or more times.

In conclusion, early diagnosis of breast cancer by screening reduces breast cancer mortality in the longer term. Two forms of bias due to the case–control study design seem to influence the protective effect in different directions; the overall bias probably results in a small overestimation of the overall protective effect. The choice of a 2-yearly interval in the nationwide Dutch screening programme is supported by the results of the present study.

### ACKNOWLEDGEMENTS

The authors thank Mrs AC Hekking, Mrs WC Schouten, BJ Slotboom, Mrs IDM Thielen, and Mrs MJH Rollema-Stekelenburg for their assistance by collecting and processing the data. Thanks are also extended to the Central Bureau of Statistics, general practitioners, local authorities, and the 'Stichting Preventicon voor de Vroege Opsporing van Borstkanker in Midden-Nederland' for their cooperation. This study was funded by the Praeventiefonds and the National Health Insurance Board.

### REFERENCES

Asbury D, Boggis CRM, Sheals D, Threlfall AG and Woodman CBJ (1996) NHS breast screening programme: is the high incidence of interval cancers inevitable? *BMJ* 313: 1369–1370

Breslow NE and Day NE (1980) The analysis of case–control studies. In *Statistical Methods in Cancer Research*, Davies W (ed.), pp. 162–189. IARC Scientific publications no 32. IARC, Lyon

 © Cancer Research Campaign 1998

Collette HJA (1985) Attempts to evaluate a non-randomized breast cancer screening programme (the DOM project). *Maturitas* **7**: 43–50

Collette HJA. Day NE. Rombach JJ and de Waard F (1984) Evaluation of screening for breast cancer in a non-randomised study (the DOM project) by means of a case–control study. *Lancet* **I**: 1224–1226

Collette HJA. Waard F de. Rombach JJ. Collette C and Day NE (1992) Further evidence of benefits of a (non-randomised) breast cancer screening programme: the DOM project. *J Epidemiol Community Health* **46**: 382–386

Egret (1990) Statistical package. Statistics and Epidemiology Research Corporation: Seattle

Hakama M. Holli K. Isola J. Kallioniemi OP. Kärkkäinen A. Visakorpi T. Pukkala E. Saarenmaa I. Geiger U. Ikkala J. Nieminen T. Godenhjelm K and Koivula T (1995) Aggressiveness of screen-detected breast cancers. *Lancet* **345**: 221–223

Hosek RS. Flanders WD and Sasco AJ (1996) Bias in case–control studies of screening effectiveness. *Am J Epidemiol* **143**: 193–201

Koning HJ de. Fracheboud J. Boer R. Verbeek ALM. Collette HJA. Hendriks JHCL. Ineveld van BM. Bruyn de AE and Maas van der PJ (1995*a*) Nationwide breast cancer screening in The Netherlands: support for breast cancer mortality reduction. *Int J Cancer* **60**: 777–780

Koning HJ de. Boer R. Warmerdam PG. Beemsterboer PMM and Maas van der PJ (1995*b*) Quantitative interpretation of age-specific mortality reductions from the Swedish breast cancer-screening trial. *J Natl Cancer Inst* **87**: 1217–1223

Nab HW (1995) Trends in incidence and prognosis in female breast cancer since 1955. Registry-based studies in south-east Netherlands. PhD thesis: Erasmus University Rotterdam

Patnick J. Austoker J and Wolff T (1995) Revision of 'NHS breast cancer screening: the facts': an evaluation. *J Med Screening* **2**: 15–17

Tabàr L. Fagerberg G. Chen HH. Phil M. Duffy SW. Smart CR. Gad A and Smith RA (1995) Efficacy of breast cancer screening by age. New results of the Swedish two-country trial. *Cancer* **75**: 2507–2517

Waard F de. Collette HJA. Rombach JJ. Baanders-v Halewijn and Honing C (1984) The DOM project for the early detection of breast cancer. Utrecht. The Netherlands. *J Chronic Dis* **37**: 1–44

Waard F de. Collette HJA and Rombach JJ (1986) Het DOM-project voor de vroege opsporing van borstkanker te Utrecht. deel 3. 1986

Weiss NS and Lazovich DA (1996) Case–control studies of screening efficacy: the use of persons newly diagnosed with cancer who later sustain an unfavorable outcome. *Am J Epidemiol* **143**: 319–322

© Cancer Research Campaign 1998


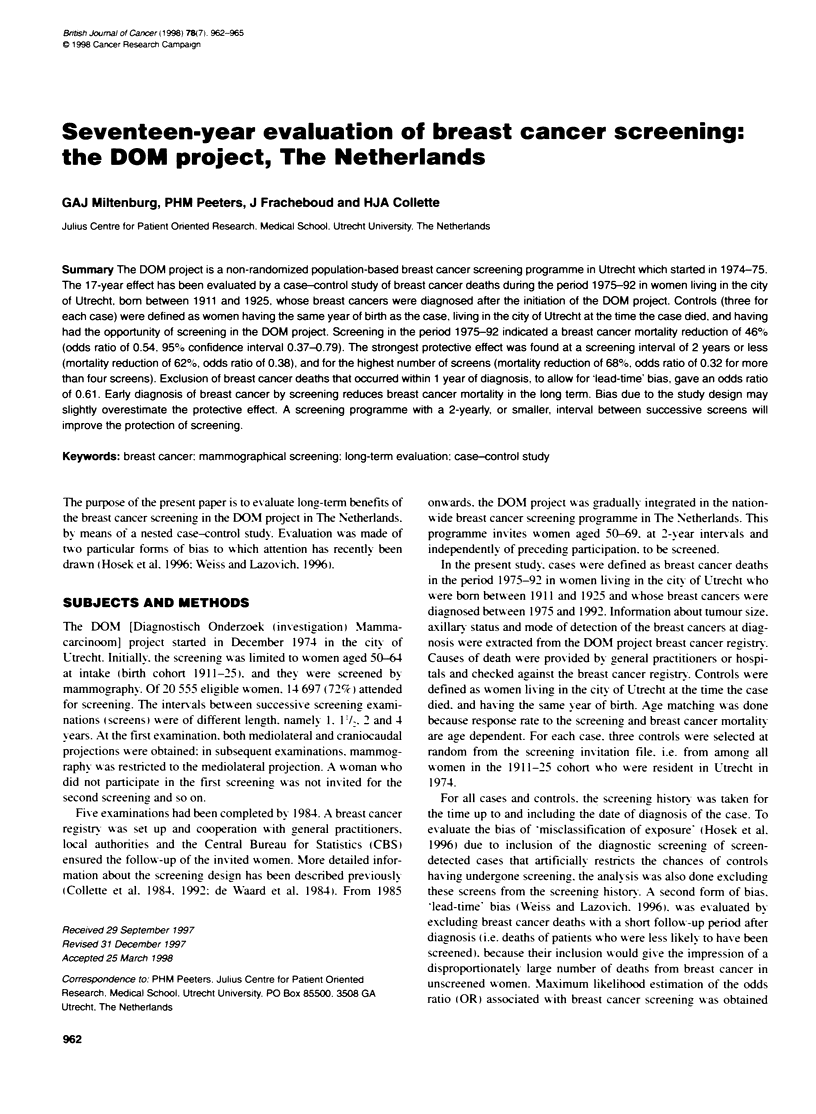

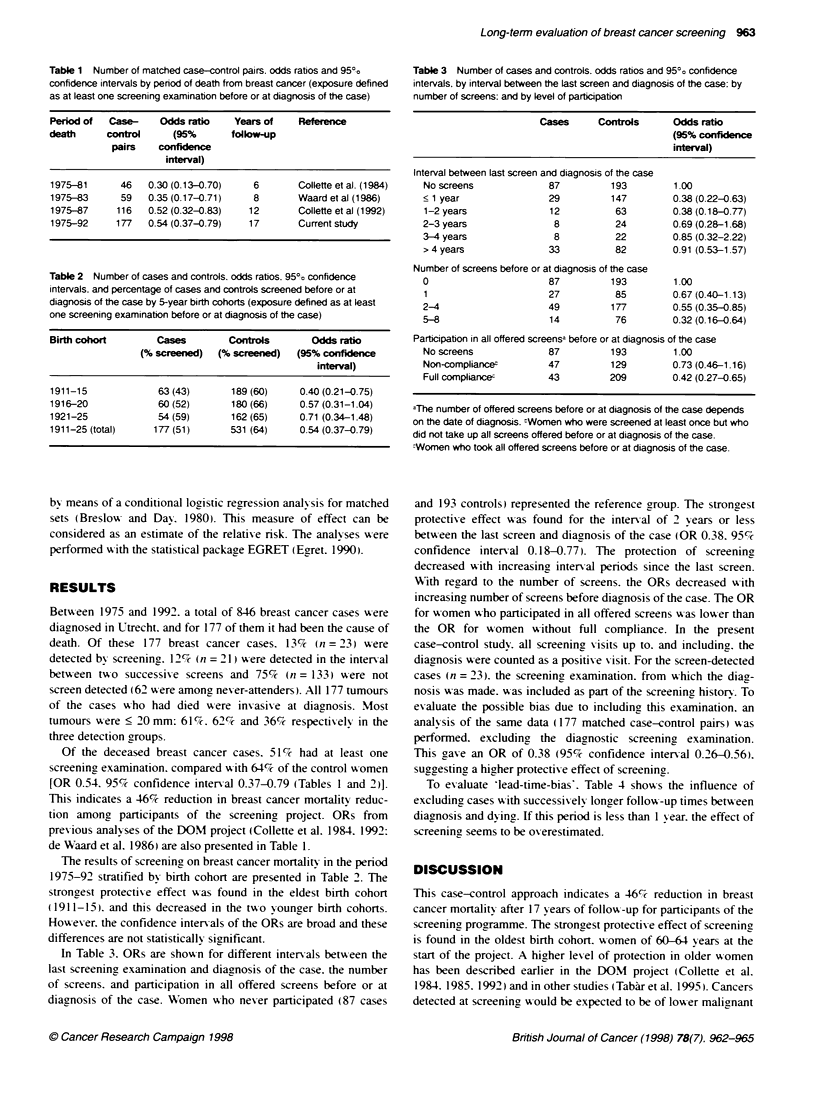

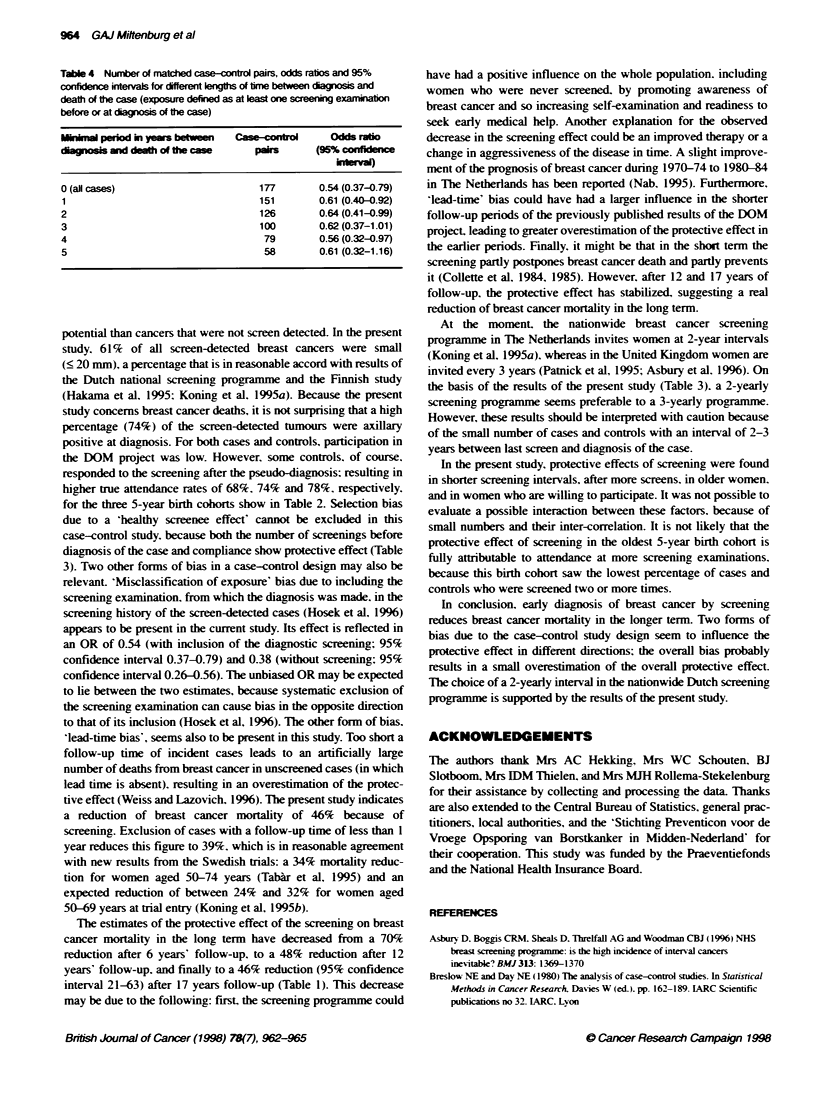

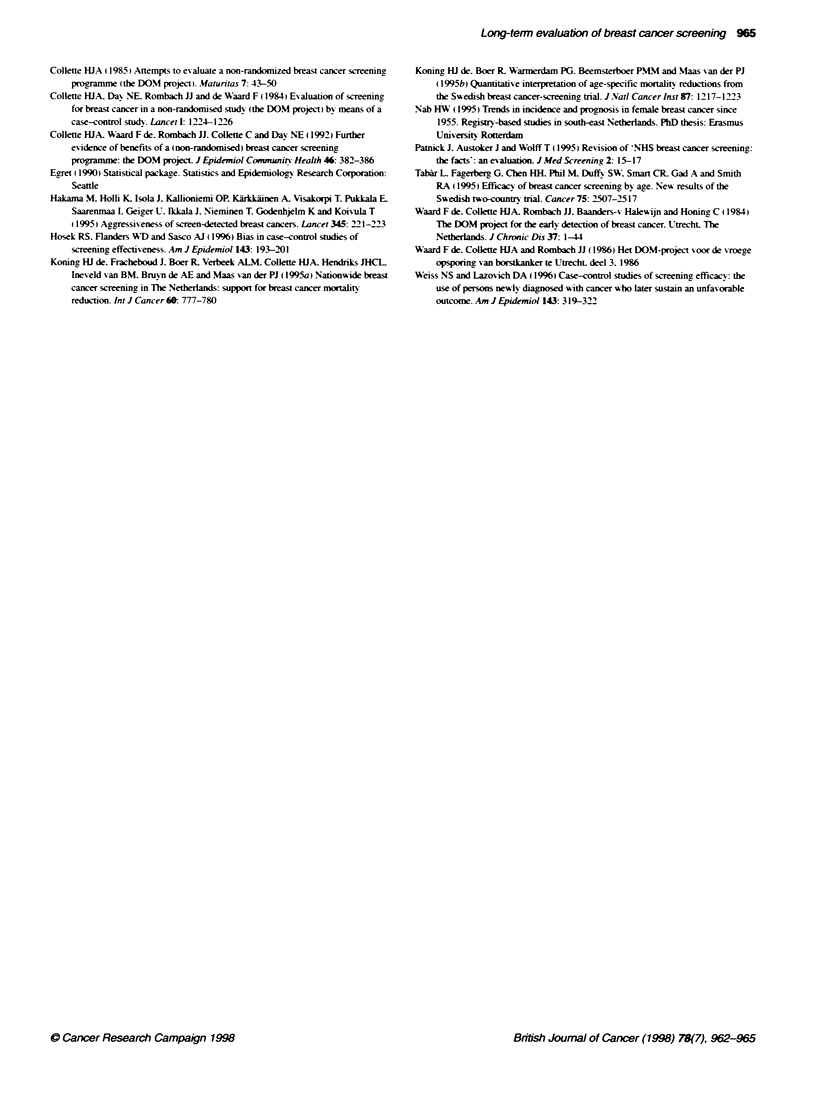


## References

[OCR_00394] Asbury D., Boggis C. R., Sheals D., Threlfall A. G., Woodman C. B. (1996). NHS breast screening programme: is the high incidence of interval cancers inevitable?. BMJ.

[OCR_00053] Collette C., Collette H. J., Fracheboud J., Slotboom B. J., de Waard F. (1992). Evaluation of a breast cancer screening programme--the DOM project.. Eur J Cancer.

[OCR_00410] Collette H. J. (1985). Attempts to evaluate a non-randomized breast cancer screening programme (the 'DOM-project').. Maturitas.

[OCR_00414] Collette H. J., Day N. E., Rombach J. J., de Waard F. (1984). Evaluation of screening for breast cancer in a non-randomised study (the DOM project) by means of a case-control study.. Lancet.

[OCR_00419] Collette H. J., de Waard F., Rombach J. J., Collette C., Day N. E. (1992). Further evidence of benefits of a (non-randomised) breast cancer screening programme: the DOM project.. J Epidemiol Community Health.

[OCR_00439] De Koning H. J., Fracheboud J., Boer R., Verbeek A. L., Collette H. J., Hendriks J. H., van Ineveld B. M., de Bruyn A. E., van der Maas P. J. (1995). Nation-wide breast cancer screening in The Netherlands: support for breast-cancer mortality reduction. National Evaluation Team for Breast Cancer Screening (NETB).. Int J Cancer.

[OCR_00432] Hosek R. S., Flanders W. D., Sasco A. J. (1996). Bias in case-control studies of screening effectiveness.. Am J Epidemiol.

[OCR_00451] Patnick J., Austoker J., Wolff T. (1995). Revision of "NHS breast screening: the facts": an evaluation.. J Med Screen.

[OCR_00455] Tabar L., Fagerberg G., Chen H. H., Duffy S. W., Smart C. R., Gad A., Smith R. A. (1995). Efficacy of breast cancer screening by age. New results from the Swedish Two-County Trial.. Cancer.

[OCR_00469] Weiss N. S., Lazovich D. (1996). Case-control studies of screening efficacy: the use of persons newly diagnosed with cancer who later sustain an unfavorable outcome.. Am J Epidemiol.

[OCR_00444] de Koning H. J., Boer R., Warmerdam P. G., Beemsterboer P. M., van der Maas P. J. (1995). Quantitative interpretation of age-specific mortality reductions from the Swedish breast cancer-screening trials.. J Natl Cancer Inst.

[OCR_00460] de Waard F., Collette H. J., Rombach J. J., Baanders-van Halewijn E. A., Honing C. (1984). The DOM project for the early detection of breast cancer, Utrecht, The Netherlands.. J Chronic Dis.

